# Mucosal T cell activation pathways are upregulated by equine herpesvirus type 1 infection

**DOI:** 10.1186/s13567-026-01741-x

**Published:** 2026-04-06

**Authors:** Camille M. Holmes, Susanna Babasyan, Bettina Wagner

**Affiliations:** Department of Population Medicine and Diagnostic Sciences, College of Veterinary Medicine, Cornell University, Ithaca, NY USA

**Keywords:** Mucosal immunity, adaptive immune response, T cell, viral infection, equine

## Abstract

Equine herpesvirus type 1 (EHV-1) is a high morbidity and mortality virus that impacts horse populations worldwide. As a respiratory virus, it enters through the upper respiratory tract (URT), where mucosal immunity plays a crucial role in preventing severe disease. In this study, flow cytometry was used to characterize the nasal leukocyte population during EHV-1 infection, and RNA sequencing of nasal secretions was employed to assess transcriptional markers of the mucosal immune response. Horses with distinct immune statuses were compared at four stages: pre-infection, early (day 1 and 3 post-infection [pi]), mid (days 8 and 10 pi) and late (day 18 pi) infection. Non-immune horses (*n* = 4), developed clinical signs, shed virus, and established viremia, whereas, immune horses (*n* = 4), possessing pre-existing immunity, showed no disease or detectable virus after EHV-1 infection. This comparison revealed differential expression of various T cell associated markers based on pre-existing immunity. In immune horses, upregulation of fewer genes occurred during early infection only, while in non-immune horses, a large number of genes were upregulated during mid and late infection. Collectively, these findings highlight a key role for mucosal T cells in mediating viral clearance and restoring homeostasis.

**Supplementary Information:**

The online version contains supplementary material available at 10.1186/s13567-026-01741-x.

## Introduction

The airways are constantly exposed to environmental microbes, provide an entry site for many respiratory pathogens, and represent one of the body’s major mucosal surface areas for host immunity to prevent and regulate disease [1–4]. In mammals, the respiratory tract is divided into the upper respiratory tract (URT), which warms and humidifies air, and filters debris [5], and the lower respiratory tract (LRT), where oxygen exchange occurs [6, 7]. Respiratory viruses spread through several transmission routes, including direct contact, fomites, droplets, and aerosols [8]. Ease of transmission can allow for rapid spread within a population, complicating disease control efforts. The size of respiratory particles impacts the location of deposition, with larger particles depositing in the URT, and smaller particles reaching the LRT [9]. The URT serves as the primary entry site for many viruses and mucosal immunity at the URT is a crucial first line of defense against infection, influencing disease severity, viral spread, and disease outcomes.

In previously exposed individuals, immune memory is established, and immune cells and components can home to mucosal tissues, enabling rapid localized protection upon re-exposure to respiratory viruses. Mucosal antibodies (mucAbs), secreted by localized plasma cells, are transported to the mucosal surfaces to neutralize pathogens and block viral entry [10, 11]. Dimeric IgA and monomeric IgG are the prominent mucosal isotypes and cross the epithelium via polymeric immunoglobulin receptors (pIgR) and neonatal Fc receptors (FcRn), respectively [12–16]. In humans, mucosal T cells are the predominant immune cell population in the epithelium, with an estimated one intraepithelial T cell (T-IEL) for every nine epithelial cells, in healthy tissue [17]. These T cells can orchestrate immune responses by producing cytokines and chemokines or by eliminating virally infected cells through cytotoxic granule release. Together, mucAbs and T-IELs can effectively inhibit viral entry and replication, thereby limiting disease progression.

Similar to humans, horses experience seasonal respiratory viral outbreaks. Four genera of globally distributed viruses cause morbidity and mortality in equids: influenza A viruses, arteritis viruses, enteroviruses, and herpesviruses [18]. Equine herpesvirus type-1 (EHV-1) is of particular concern due to the severity of clinical disease and its ability to establish latency. In non-immune horses, EHV-1 replicates in the URT during the early stage of infection, producing clinical signs such as fever, nasal and ocular discharge, lethargy, and depression [19]. As the infection progresses, immune cells in the URT may become infected, facilitating viral trafficking to the lymph nodes, and establishing a cell-associated viremia [20–24]. In some infected horses, viral replication in the vasculature can induce thrombo-ischemia in the central nervous system or placenta, causing neurological disease or abortion, respectively [25, 26].

EHV-1 immune horses have pre-existing mucAbs specific to the EHV-1 glycoproteins essential for cell entry, including glycoprotein B, C, and D (gB, gC, and gD) [27–29]. The mucAb response, dominated by IgG1 and IgG4/7, neutralizes cell-free virus, preventing viral entry at the primary site of infection and mitigating disease [27]. EHV-1 specific T cells can be recovered from the draining lymph nodes and peripheral blood of horses [30–36], but their presence in mucosal tissues remains uncertain. In healthy horses, CD4^+^ and CD8^+^ T cells, of likely multiple antigen-specificities, are the dominant immune cell population at the URT and can be visualized in the mucosa and submucosa by microscopy [37–39]. EHV-1 infection provokes an increase in leukocyte numbers and a shift in the T cell ratio towards an increase in CD8^+^ T cells [37]. Together this suggests a role for mucosal T cells in immunity after EHV-1 infection.

This study investigates the dynamics of mucosal immune responses following internasal challenge with EHV-1. Horses were classified as non-immune, if clinical disease and viral replication were detected, or immune, if pre-existing mucAb were present and clinical disease and viral replication were absent after challenge infection. Intranasal leukocyte populations were characterized in nasal washes by flow cytometric analysis, and nasal gene expression was analyzed by RNA sequencing (RNA-seq) of nasal swab samples. This data provides insights into differences in mucosal immune responses based on immune status and time of infection, with a focus on mucosal T cell functions.

## Materials and methods

### EHV-1 infection of horses

All samples were collected during an experimental EHV-1 infection, which has previously been described in detail [28]. In brief: horses were individually housed in stalls with no direct contact with other horses, and free access to water, salt blocks, and grass hay. They were intranasally challenged with 1 × 10^7^ PFU neuropathogenic EHV-1 Ab4. The immune status of the horses as immune, partially immune, or non-immune was determined retrospectively, after analyzing all viral, clinical, and serological parameters after challenge infection as previously described [28]. Non-immune horses did not have pre-existing intranasal or systemic EHV-1 specific IgG4/7, developed fever after EHV-1 infection, shed high amounts of virus in their nasal secretions, and developed cell-associated viremia (Table 1, Additional file 1). All non-immune horses were previously infected with EHV-1 two or more years prior to this experimental challenge. Immune horses had pre-existing intranasal and systemic EHV-1 specific IgG4/7, no fever, and neither nasal viral shedding nor cell-associated viremia were detected (Table 1, Additional file 1). Partially immune horses had an intermediate phenotype without clinical signs and some low, short-lasting viral shedding and/or cell-associated viremia. Immune and partially immune horses were last infected with EHV-1 9 months prior to this experimental challenge infection. For nasal cell characterization by flow cytometry, the non-immune group consisted of eight horses (4 geldings, and 4 mares) and the other group was composed of immune and partially immune horses (8 geldings, and 8 mares), that were 3–5 years of age. For RNAseq, four immune and four non-immune horses were used, and each group consisted of 3 geldings and 1 mare that were 3–4 years of age.

**Table 1:** **EHV-1 immune status based on clinical evaluation of horses and viral detection**

Horse information^a^		Highest body temperature^b^		Highest nasal shedding^c^		Highest viremia^d^		EHV-1 specific IgG4/7^e^	
EHV-1 immune status	ID	°C	Hour pi	PFU/mL	Day pi	Ct	Day pi	Intranasalpre/d1pi (MFI)	Serum pre/d1pi (MFI)
Non-immune horses	3	41.2	36	19500	3	31.25	6	2/2	3/3
	5	42.4	48	700000	2	30.43	4	5/3	36/39
	15	41.6	48	240000	2	33.71	5	2/2	11/10
	20	41.4	48	52500	2	31.32	7	2/3	7/8
Immune	6	38.3	12	ND	ND	ND	ND	93/993	4783/5124
	11	38.0	12	ND	ND	ND	ND	7/53	4654/3786
	14	38.1	12	ND	ND	ND	ND	27/234	1134/838
	24	38.3	72	ND	ND	ND	ND	75/1077	3906/3314

All horses were intranasally infected with 1 × 10^7^ PFU neuropathogenic EHV-1 Ab4 and monitored daily for clinical signs of disease.

ND: not detected, MFI: median fluorescence intensity, PFU: plaque-forming units.

^a^Immune status was determined following infection based on presence of fever and viral detection.

^b^Body temperatures measured by rectal temperature; fever was defined as body temperature of 38.6 °C or higher.

^c^Nasal shedding was measured in nasal secretions by plaque assay.

^d^Cell associated viremia was measured in PBMC by EHV-1 specific qPCR.

^e^Intranasal and systemic EHV-1 specific IgG4/7 antibodies were measured by a fluorescent bead-based assay in nasal secretions and serum pre-infection and on d1 pi.

All horse procedures were approved by the Institutional Animal Care and Use Committee at Cornell University (protocol #2011-0011). Additionally, they were carried out by the recommendation in the *Guide for the Care and Use of Laboratory Animals* of the National Institutes of Health and *Guide for Care and Use of Animals in Agricultural Research and Teaching.*

### Laboratory testing to support the determination of each horse’s EHV-1 immune status

The EHV-1 immune status of each horse was supported by the presence or absence of fever after infection and by the following laboratory methods: Nasal shedding was determined by virus isolation using a plaque assay as previously described [27–29, 40, 41]. The plaque assay measured replicating virus. The correlation of detecting replicating virus in nasal secretions in comparison to DNA detection by real-time PCR was recently published for EHV-1 immune and non-immune horses [27]. Cell-associated viremia was detected by real-time PCR of the EHV-1 gB gene and was performed at the Animal Health Diagnostic Center (AHDC) at Cornell University. Nasal mucosal and serum EHV-1 specific IgG4/7 antibodies were quantified in the EHV-1 Risk Evaluation assay at the AHDC a using a fluorescent bead-based assay that was previously described in detail for both serum and mucosal sample [27–29, 40–43].

### Intranasal cell collection, processing, and extracellular staining for flow cytometry

Intranasal cells were collected by nasal washes, before and after EHV-1 Ab4 challenge, using 50 mL of sterile saline, which was intranasally administered with a catheter (12fr × 16inch sterile urethral catheter, Cardinal Health, Dublin, OH, USA) to flush the nasal cavity. The wash fluid was recovered in a clean plastic bag positioned under the horse’s nose. Samples were centrifuged at 300 × *g* for 5 min to pellet cells, supernatants were removed, cells were resuspended in sterile PBS, and pooled by group, non-immune and immune/partially immune, respectively, to ensure adequate cell numbers for flow cytometry analysis. Next, intranasal cells were isolated for each group by density centrifugation (Ficoll-Paque PLUS; GE Healthcare, Piscataway, NJ, USA). Isolated cells were washed once in PBS, fixed in 2% formaldehyde for 20 min at room temperature, washed twice in PBS afterwards, and then resuspended in PBS/BSA (PBS with 0.5% bovine serum albumin (Thermo Fisher Scientific, Waltham, MA, USA). Fixed cells were triple stained for the leukocyte marker LFA-1 (cz3.2) [48], CD4 (HB61A) [49], and CD8 (CVS8) as previously described [50]. All cells were measured in a FACSCanto II flow cytometer (BD Biosciences, San Diego, CA, USA) and analyzed using FlowJo version 10.8.1 (FlowJo, Ashland, OR, USA). A minimum of 10 000 events per sample were recorded.

### Nasal swabs, RNA extraction, and RNA sequencing

Nasal secretion samples were taken throughout infection as previously described [27]. Briefly, two polyester tipped applicators (Puritan Medical Products Company, Gullford, Maine, USA) were inserted into the nose and rotated against the mucosa for about five seconds. One of the swabs was stored in a tube with no additive at -80 ºC until processing for bulk RNA sequencing. Swabs were taken from immune (*n* = 4) and non-immune (*n* = 4) horses 2 days prior to infection (d-2) and on day 1 post-infection (pi), day 3 pi, day 8 pi, day 10 pi, and day 18 pi, processed and sequenced as previously described [27]. Quality control checks were utilized to exclude samples with low host gene expression. The remaining samples offered coverage of both the non-immune and immune groups throughout viral infection.

### RNAseq analysis of host gene expression

Sequence data was analyzed using integrated Differential Expression and Pathway analysis (iDEP.951), as previously described [44]. Samples were grouped based on the immune status of the horse and time of infection: pre (day-2), early (day 1–3 pi), mid (day 8–10 pi) and late (day 18 pi). Raw read counts were uploaded and annotated with the equine genome assembly (EquCab3.0). Counts were normalized using a variance stabilizing transformation (VST). Differential gene expression analysis based on the negative binomial distribution (DEseq2) [45] was used to determine differentially expressed genes (DEGs), based on the combination of immune status and/or infection timepoint. The cut-off value for significance was set at adjusted *p*-value (p-adj) < 0.05 and for fold change was set at abs(log_2_FC) ≥ 1.0. Distribution of all genes in the dataset was visualized in a volcano plot, graphing log_2_FC by p-adj. To determine if DEGs were functionally involved in the immune response, g:Profiler (version e112_eg59_p19_25aa4782) was used [46]. The equine genome assembly (EquCab3.0, GenBank accession: GCA_002863925.1) was used for reference, and DE genes within the Gene Ontology Biological Process (GO:BP) term “immune system process” (GO:0002376), “innate immune response” (GO:0045087), “adaptive immune response” (GO:0002250), “T cell activation” (GO:0042110), “regulation of metabolic process” (GO:0019222), “regulation of gene expression” (GO:0010468), “regulation of biosynthetic process” (GO:0009889), were determined. Genes within the T cell activation pathway were visualized by heatmaps, with group specific pre- vs post-infection Log_2_FC determining the intensity of coloration and DEseq2 results for immune vs non-immune expression determining the displayed p-values. The specific function of each gene was classified by literature review and genes were sub-grouped based on their role in T cell activation.

## Results

### Flow cytometric analysis of intranasal T cells during EHV-1 infection

**Figure 1:**
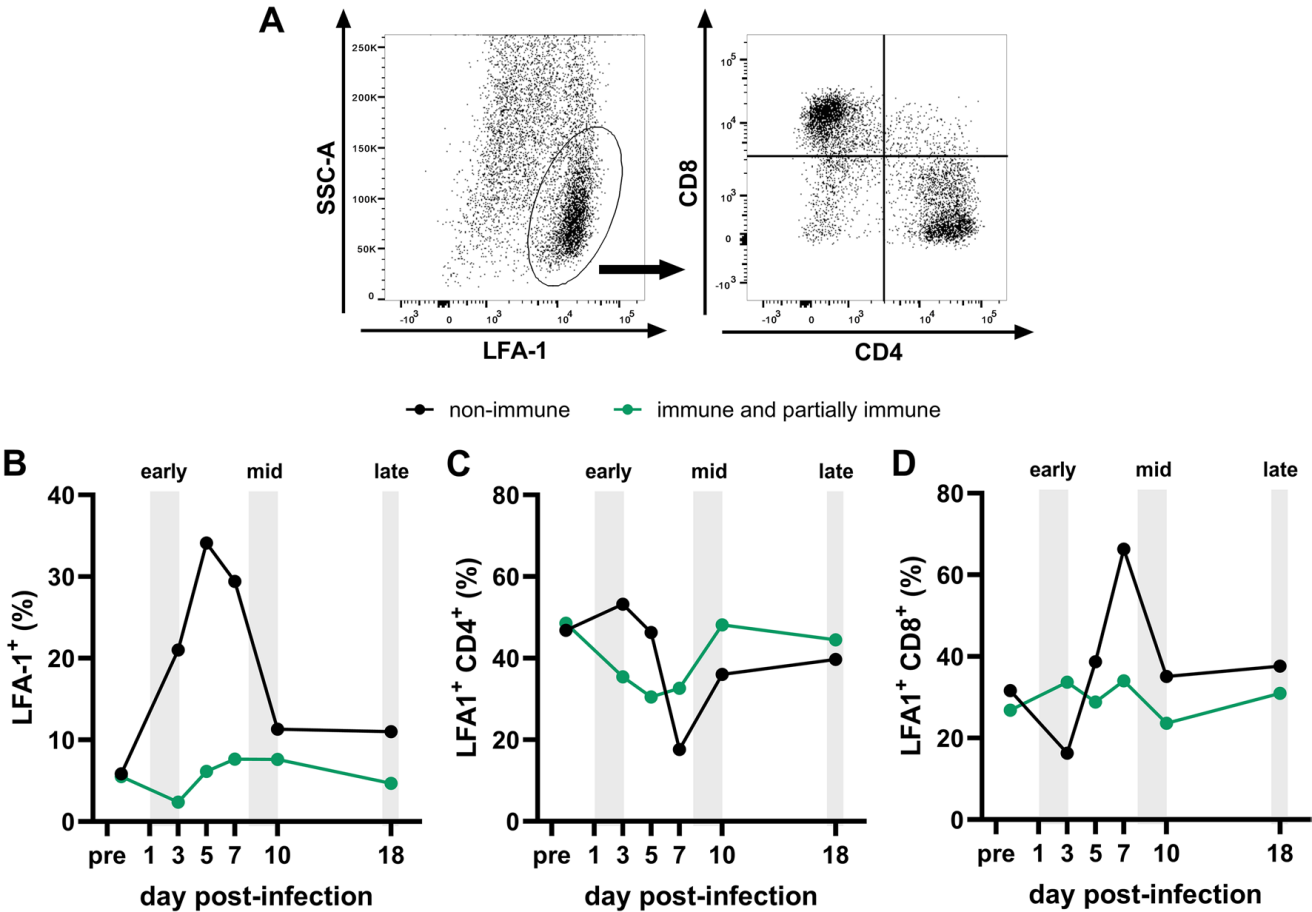
**Intranasal T cell phenotype dynamics during EHV-1 infection.** Horses were either immune or partially immune (green, n = 16) following prior infection with EHV-1 vaccine candidate strains or wildtype Ab4 strain, or non-immune (black, n = 8). All horses were intranasally challenged with the Ab4 strain of EHV-1. Nasal washes were collected throughout the infection and pooled by group for intranasal immune cell phenotyping. Cells were triple-stained with LFA-1 (to distinguish epithelial cells leukocytes) and with CD4 and CD8 (to identify T cells). **A** A representative gating strategy for the non-immune group is shown. The percentages of **B** LFA-1^+^ leukocytes, and **C** CD4^+^ T cells and **D** CD8^+^ T cells within the LFA-1^+^ leukocyte population are displayed.

Horses with or without immunity from prior EHV-1 infection were challenged with the Ab4 strain of EHV-1. Nasal cells were collected via nasal washes throughout infection to assess immune cell population dynamics. LFA-1 expression differentiated leukocytes from structural cells, and CD4 and CD8 markers identified T cell populations within the LFA-1^+^ population (Figure 1A). Pre-infection leukocyte percentages were unaffected by pre-existing immunity, comprising approximately 5% of nasal cells (Figure 1A). Following EHV-1 challenge, immune/partially immune horses exhibited minimal changes in leukocyte percentages. Conversely, non-immune horses had an influx of leukocytes peaking with about 35% at day 5 pi (Figure 1B). T cells constituted the predominant intranasal immune population throughout infection, comprising 65–85% of LFA-1^+^ cells (Figures 1C, D). Immune/partially immune horses had a transient decrease in CD4^+^ T cell percentages during the first 5 days pi, returning to baseline levels by the end of the week (Figure 1C), while their CD8^+^ T cell population had minor fluctuations throughout infection (Figure 1D). In contrast, non-immune horses had a clear decrease in CD4^+^ T cell percentages to about half of the pre-infection percentages by day 7 pi, which corresponded to an increase in CD8^+^ T cell percentages on the same day (Figures 1C, D). Overall, intranasal leukocyte percentages and CD4/CD8 T cell ratios stayed relatively constant after EHV-1 challenge in immune horses. Non-immune horses experienced a 6–sevenfold increase in their intranasal leukocyte percentages between days 3 and 7 pi compared to pre-infection, together with a shift towards CD8^+^ intranasal T cells towards the end of the first week post EHV-1 infection.

### Differential nasal gene expression pre- and post EHV-1infection

**Figure 2:**
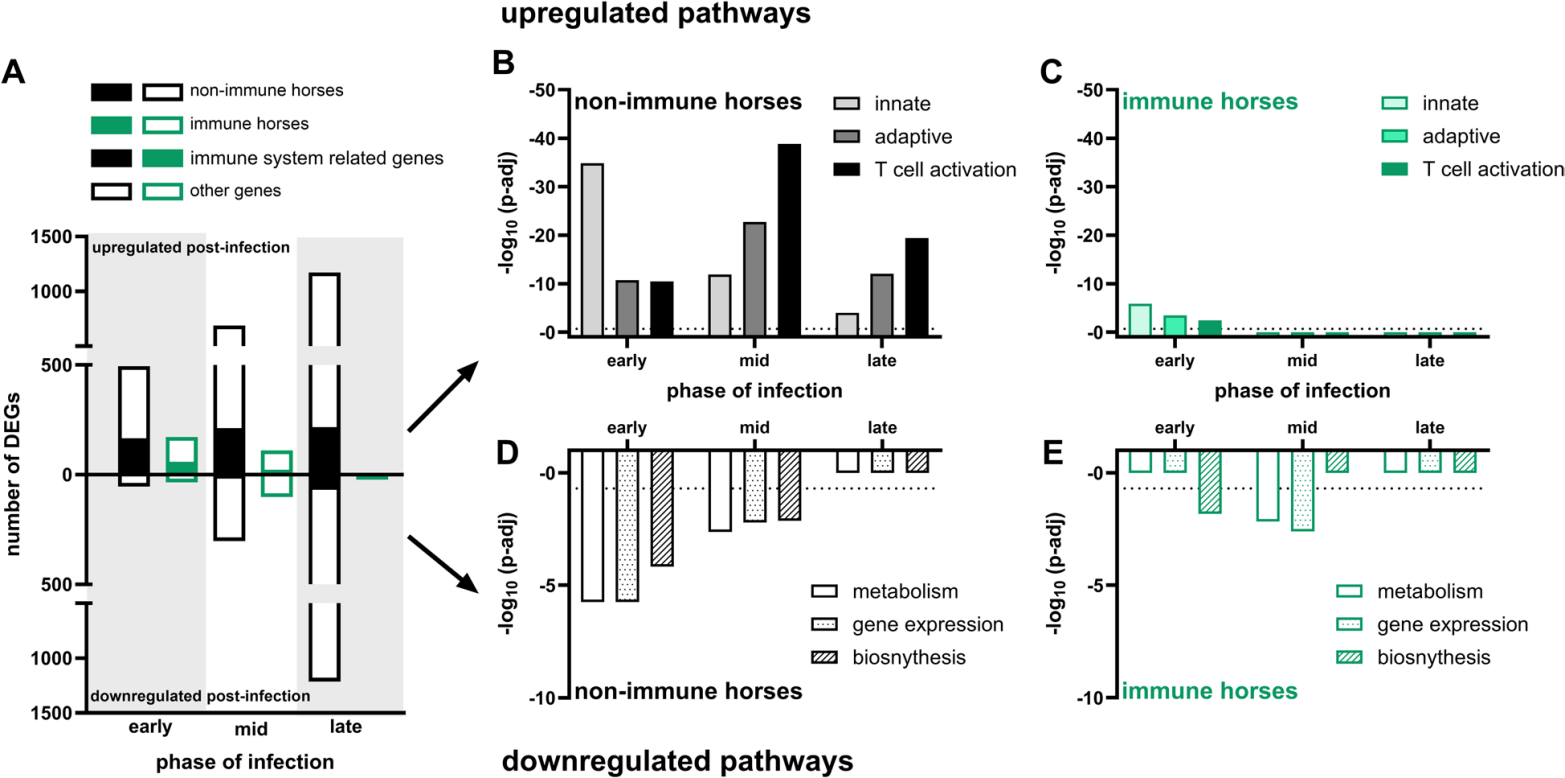
**Nasal mucosal gene expression profiles pre- and post-EHV-1 infection.** *RNA* sequencing was performed on nasal swabs collected from immune (*n* = 4, green) and non-immune (*n* = 4, black) horses. Immune status was retrospectively determined based on the presence or absence of clinical signs and virus detection in nasal secretions and peripheral blood of the horses. DEGs pre- and post-infection were identified using DEseq2. Cut-off values were set at abs(log_2_FC) ≥ 1.0 and p-adj < 0.05. **A** The number of DEGs pre-infection and during early (left), mid (middle), and late (right) infection is shown, with upregulated genes depicted above the axis and downregulated genes below. Solid bars represent immune response-related genes, while open bars represent genes involved in other biological processes. Significant **B**, **C** upregulation and **D**, **E** downregulation of genes within the Gene Ontology Biological Process (GO:BP) pathways were identified using gProfiler for **B**, **D** non-immune and **C**, **E** immune horses. Data are presented as −Log_10_ values, with the horizontal dotted line at p-adj = 0.05 indicating the significance threshold for pathway dysregulation. Figure keys used the following abbreviations for defined GO:BP: immune system genes = “immune system process” (GO:0002376), innate = “innate immune response” (GO:0045087), adaptive = “adaptive immune response” (GO:0002250), T cell activation = “T cell activation” (GO:0042110), metabolism = “regulation of metabolic process” (GO:0019222), gene expression = “regulation of gene expression” (GO:0010468), and biosynthesis = “regulation of biosynthetic process” (GO:0009889).

RNAseq was performed on mRNA isolated from nasal swab samples from four immune and four non-immune horses before and after experimental challenge with EHV-1 to identify DEGs involved in the mucosal immune response. Pre- versus post-infection comparisons revealed distinct patterns of gene upregulation between immune and non-immune horses (Figure 2A).

Non-immune horses had a progressive increase in the number of upregulated genes over the course of infection: 495 genes during early infection (day 1–3 pi), 690 genes during mid infection (day 8–10 pi), and 1172 genes during late infection (day 18 pi). In contrast, immune horses upregulated the highest number of genes during early infection (172 genes), followed by fewer genes during mid infection (111 genes), with levels returning to baseline by late infection (3 genes). Upregulated genes in both groups included immune-related genes as well as genes unrelated to immune pathways. Immune-related genes accounted for 33.5% (early), 30.7% (mid), and 18.5% (late) of upregulated genes in non-immune horses, and 34.3% (early) and 19.8% (mid), in immune horses.

Pathway analysis revealed functional roles of the upregulated DEGs, identifying significant enrichment in the innate immune response, adaptive immune response, and T cell activation pathways. In non-immune horses, innate immune response gene upregulation peaked during early infection and declined thereafter, while adaptive immune response and T cell activation peaked during mid infection, with T cell activation being the most significantly upregulated (Figure 2B). In immune horses, these pathways were upregulated only during early infection and to a much lesser extent than in non-immune horses (Figure 2C).

Both groups also exhibited gene downregulation post-infection (Figure 2A). Non-immune horses downregulated the fewest genes during early infection (54 genes), followed by more during mid infection (302 genes), and the most during late infection (1213 genes). Immune horses displayed minimal gene downregulation during early (35 genes) and late infection (23 genes), with a peak during mid infection (101 genes). Downregulated genes were largely unrelated to immune pathways, comprising only 4.5–9.3% of the total. Pathway analysis highlighted disruptions in cellular functions, such as metabolism, gene expression, and biosynthesis. These perturbations were less significant compared to immune-related pathways. In non-immune horses, the significance of these pathways declined as infection progressed (Figure 2D), while immune horses showed significant downregulation only during mid infection (Figure 2E).

In summary, non-immune horses had many DEGs which continuously increased throughout infection. In non-immune horses, the upregulated genes were involved in immune response specific pathways, with days 1–3 pi dominated by innate immune responses, and a later shift to adaptive immunity by day 8–18 pi. T cell activation was the most notable immune pathway, in agreement with the shift in intranasal leukocyte populations. Meanwhile, immune horses had fewer DEGs than non-immune horses, DEGs declined as time progressed, and returned to pre-infection baseline by day 18 pi.

### Differential gene expression at the URT of immune and non-immune horses

**Figure 3:**
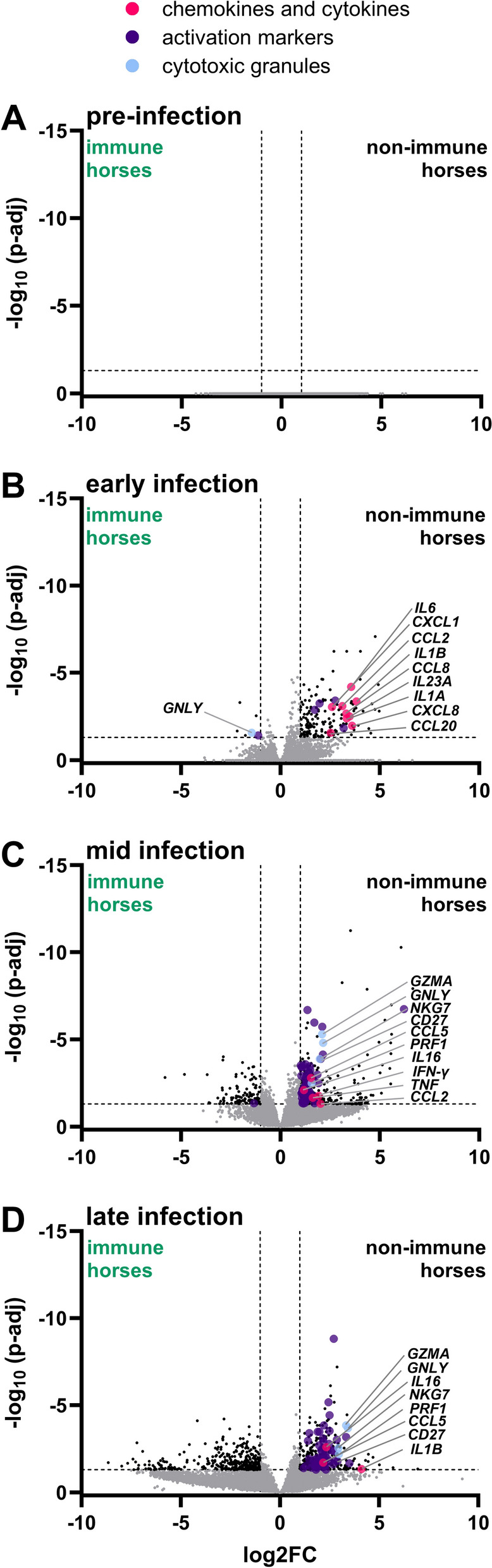
**Differential nasal gene expression in non-immune versus immune horses during EHV-1 infection.** DEGs were identified between immune (left) and non-immune (right) horses at each phase of infection using DEseq2. Volcano plots illustrate group-specific LogFC against p-adj **A** pre-infection, and during **B** early, **C** mid, and **D** late infection. P-adj is displayed as -Log_10_ values and dotted lines indicate the thresholds for significant differential expression (abs(Log_2_FC) > 1.5 and P-adj < 0.05). Black dots represent all upregulated annotated genes, while genes associated with T cell activation are highlighted: chemokines and cytokines (pink), cytotoxic granules (blue), and activation markers (purple). Selected gene names are labeled.

Gene expression profiles in immune and non-immune horses were compared at each timepoint after EHV-1 challenge to identify DEGs associated with pre-existing immunity. Pre-infection, nasal gene expression showed no significant differences between immune and non-immune horses (Figure 3A). However, distinct variations emerged during early, mid, and late infection phases. During early infection, 177 DEGs were identified: 6 genes showed higher expression in immune horses, while 171 genes displayed higher expression in non-immune horses (Figure 3B). By mid infection, 480 DEGs were detected, with 115 genes exhibiting higher expression in immune horses and 365 genes in non-immune horses (Figure 3C). During late infection, 795 DEGs were observed, with 392 genes expressed more highly in immune horses and 403 genes in non-immune horses (Figure 3D). Immune-related genes comprised approximately 34% of DEGs across all infection phases in non-immune horses. In immune horses, higher expression of immune-related genes was predominantly limited to early infection (4 out of 6 genes), while fewer than 5% of mid and late-phase DEGs were associated with immune pathways. Non-immune horses demonstrated a notable enrichment of T cell-related genes within upregulated DEGs, including markers of activation, cytokines, chemokines, and cytotoxic granules (Figures 3B–D). Overall, non-immune and immune horses had significantly different changes in mucosal gene expression in response to EHV-1 infection. Non-immune horses upregulated many immune related genes, meanwhile immune horses had higher expression of genes that were largely unrelated to immunity.

### Nasal gene expression related to T cell activation during EHV-1 infection

**Figure 4:**
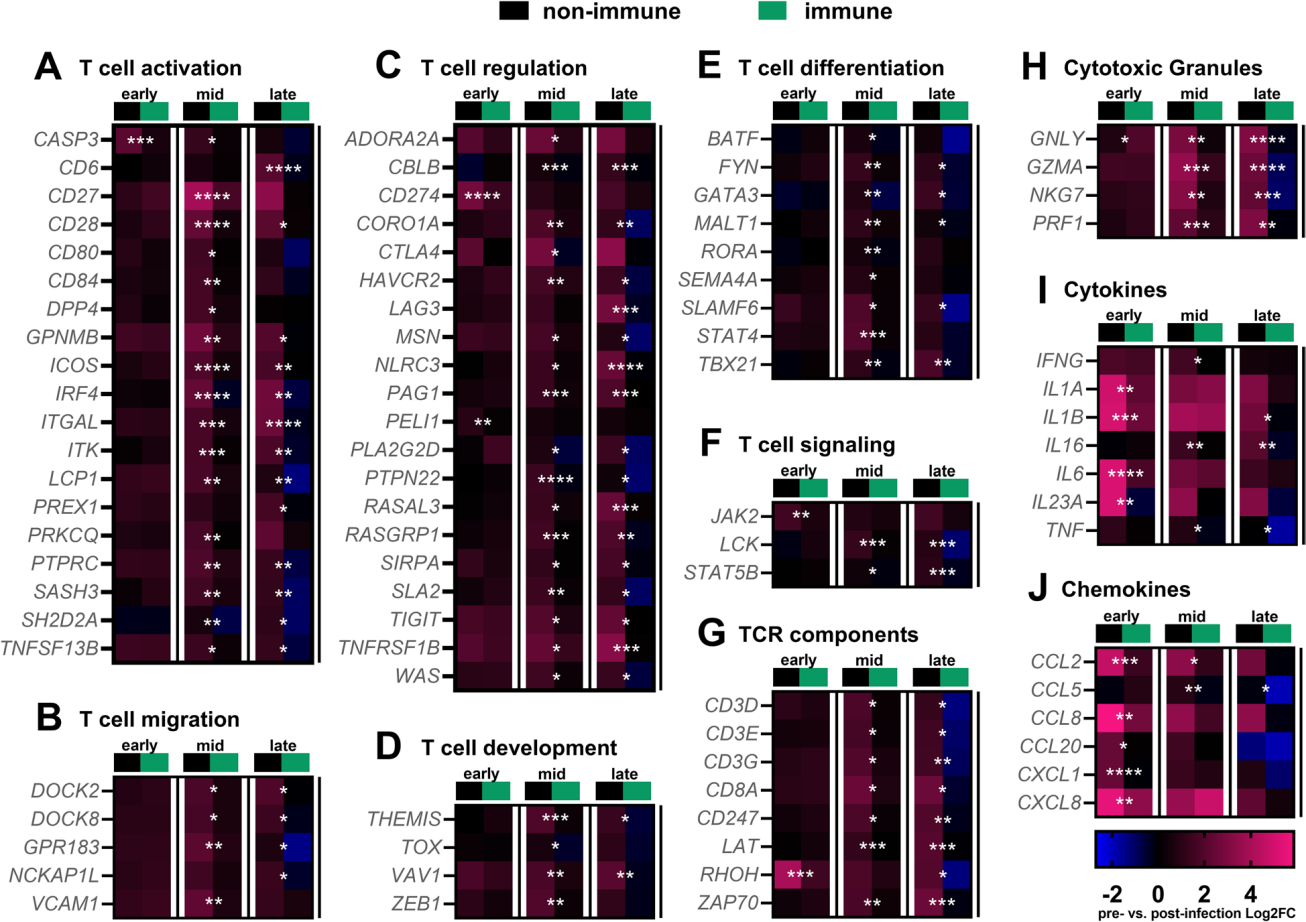
**Gene expression dynamics at the URT in immune and non-immune horses throughout EHV-1 infection.** DEGs in non-immune (black) versus immune (green) horses were identified at each phase of infection using DEseq2, focusing on genes within the T cell activation pathway. Genes were further categorized based on their role in T cell function. **A**–**J** Heat maps depict the mean Log_2_FC of gene expression from pre-infection to post-infection for each group. Blue indicates downregulation (negative Log_2_FC), pink indicates upregulation (positive Log_2_FC), and black represents minimal change in expression (Log_2_FC near zero). Genes are organized by rows and time post-infection is organized by columns for early (left), mid (center), and late (right). Comparisons between immune and non-immune horses were conducted using DEseq2, with *p*-values displayed in white for each row. *p* < 0.05 (*), *p*< 0.01 (**), *p *< 0.001 (***), and *p* < 0.0001 (****).

The comparative analysis of nasal gene expression based on immune status and infection phases identified T cell activation as a significantly upregulated pathway after EHV-1 infection of non-immune horses. DEGs within the T cell activation pathway were categorized by their roles in T cell function, including activation, migration, regulation, development, differentiation, and signaling (Figures 4A–F). Similarly, components of the T cell receptor complex, including all four chains of the CD3 complex (*CD3D, CD3E, CD3G,* and *CD247*), showed upregulation during mid and late infection (Figure 4G). Many genes associated with these functions were upregulated in non-immune horses during the mid and late phases of infection (Figures 4A–F), and a few genes were upregulated during early infection, including *CASP3*, *CD274*, *PEL1*, *JAK2*, and *RHOH* (Figures 4A, C, F, and G).

Cytotoxic granules, cytokines, and chemokines also have important roles in T cell function and exhibited similar changes in expression (Figures 4H–J). The cytotoxic granule *GNLY* displayed a distinct expression pattern, being upregulated in immune horses during early infection, and in non-immune horses during mid and late infection (Figure 4H). Other cytotoxic granule genes, *GZMA, NKG7,* and *PRF1,* were exclusively upregulated in non-immune horses and during mid and late infection (Figure 4H). Cytokine and chemokine genes were broadly upregulated in both immune and non-immune horses during early and mid infection phases, but their expression was significantly higher in non-immune horses during early infection (Figures 4I, J). The DEGs included cytokines and chemokines that are endogenously produced by T cells and those produced by other cell types and involved in orchestrating T cell migration and function.

In summary, genes related to T cell function represented a major subset of DEGs during EHV-1 infection. Changes in mucosal T cell gene expression were most notable in non-immune horses, while immune horses returned to baseline in a shorter period of time and with fewer DEGs overall. This aligns with the greater fluctuation in intranasal leukocyte numbers in non-immune compared to immune horses (Figure 1).

## Discussion

**Figure 5:**
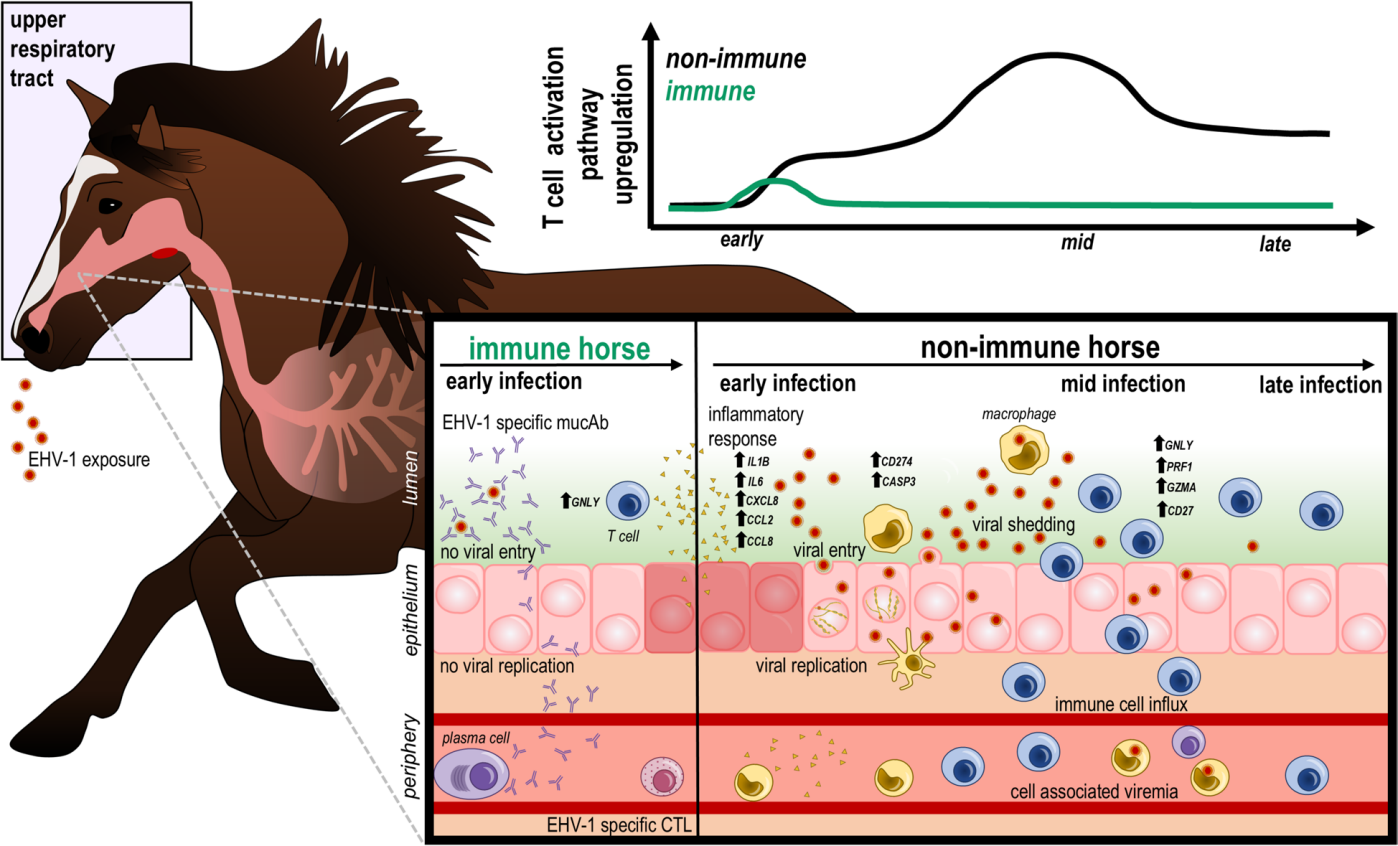
**Mucosal T cell activation during EHV-1 infection is dependent on pre-existing immunity.** Horses were intranasally challenged with EHV-1 (red virions) and viral replication at the mucosal surface (pink cells) was dependent on pre-existing immune status. Immune horses (green, right) have detectable mucAbs (purple), while non-immune horses did not (black, left). Both groups upregulated cytokines and chemokines (yellow triangles) during early infection. Immune horses upregulated the T cell (blue cells) activation pathway during early infection, while non-immune horses upregulated this pathway during mid and late infection. Selected DEGs are included besides black arrows.

EHV-1 is a prevalent pathogen in the horse population, capable of causing severe disease in the absence of prior immunity. Upon primary infection of the URT, the mucosal immune response is rapidly activated within days of exposure. While prior studies have characterized mucosal innate and adaptive response through analysis of proteins in nasal secretions, including inflammatory cytokines [28, 29, 47] and EHV-1 specific mucAbs [27–29, 40–43], cellular immunity is less well understood. Previous work from our group demonstrated that T cells are a major leukocyte population present in the nasal epithelium and also the immune cell population that increases most dramatically in the URT after EHV-1 infection [37], which suggests that T cells are well positioned for rapid response upon infection. In this study, we further analyzed mucosal T responses utilizing flow cytometry and RNAseq to identify changes in mucosal immune cell populations and transcriptional profiles after EHV-1 infection, revealing a clear temporal progression of the mucosal immune response (Figure 5).

In the URT of non-immune horses, EHV-1 can freely replicate during the first days after infection [24, 40, 41]. During viral replication, leukocyte populations in the URT expanded alongside transcriptomic shifts, marked by differential expression of numerous immune-related genes. CD4^+^ and CD8^+^ T cells were the dominant leukocyte population throughout infection, and T cell related gene expression was overall the most highly upregulated pathway. These changes occurred in synchrony with the progression of infection. In the first 3 days post-infection, viral entry and initial replication were accompanied by viral shedding. This stage was dominated by innate immunity, characterized by robust upregulation and secretion of inflammatory cytokines and chemokines [28, 47], which likely contributed to the increasing number of leukocytes. Additionally, programmed death-ligand 1 (*CD274*) was upregulated while CD8^+^ T cell percentages declined, indicating a phase of immune suppression during early infection. During viral infection, CD274 (PD-L1) expression is driven by inflammation, and can be detected on epithelial and immune cells [48]. Through interactions with PD-1, PD-L1 modulates T cell activity, dampening immune responses [49–51]. In LCMV infection, PD-L1 expression on epithelial cells has been implicated in viral persistence due to reduced T cell function [52], and a comparable process may occur during early EHV-1 infection, when viral shedding reaches its peak. Single cell RNAseq of mucosal cells would provide additional clarity on differential expression of genes, such as PD-L1, that are not cell type specific and is the aim of future studies.

Between early and mid-infection, the CD4^+^/CD8^+^ T cell ratio shifted, with increasing CD8^+^ T cell percentages. This shift mirrored previous findings in non-immune horses [37]. In this study, nasal cells were analyzed in pooled nasal wash samples to achieve adequate cell numbers, which limited statistical analysis. Additional studies, using recently described mucosal sampling methods [37] can be utilized in the future to further evaluate the dynamics of leukocyte populations during EHV-1 infection on an individual horse level. Following the transition in T cell ratio, expression of T cell activation markers, such as *CD27* and *CD28*, and effector proteins, including, interferon gamma (*IFNG),* perforin (*PRF1)*, granulysin (*GNLY*), and granzyme A (*GZMA*), increased. This suggests a rebound of the T cell population and its activation, contributing to viral clearance. CD27 and CD28 are co-stimulatory receptors expressed on effector T cells that interact with CD70 and CD80, respectively, to promote T cell survival and proliferation [53]. IFN-γ is a key cytokine that orchestrates immune responses to intracellular pathogens and viral infection, and is detectable in nasal secretions and sera of infected horses [28, 29, 40, 41]. Cytotoxic granules, such as perforin, granulysin, and granzyme can target and eliminate infected cells. Although these proteins have not been thoroughly characterized in horses, their homologs in other species play essential roles in immunity, operating sequentially to form pores in infected cells and initiate programmed cell death pathways [54–56]. By mid-infection, viral shedding decreased, suggesting the initiation of T-cell mediated control in addition to the appearance of mucAb production (27–29). By late infection, clinical disease had been resolved, and virus could no longer be detected in nasal secretions or peripheral blood. During this phase, some genes returned to baseline expression levels, while others maintained elevated expression. Together, this demonstrated that innate immunity at the mucosal surface involved the rapid production of cytokines and chemokines to initiate and orchestrate anti-viral adaptive immunity, including a role of mucosal T cells to facilitate clearance of virus-infected cells at the URT.

In contrast, immune horses showed no viral replication and did not develop clinical disease. Pre-existing EHV-1 specific mucAbs correlated with immunity in this and previous studies, providing a mechanism for rapid neutralization and clearance of the virus at the URT [27–29, 40–43]. Nevertheless, intranasal EHV-1 exposure still transiently activated the mucosal immune response in immune horses, with the co-occurrence of innate and adaptive immunity within 1–3 days of viral challenge. This response included brief expression of selected cytokines and chemokines, previously detected in nasal secretions [28, 29], and increased expression of T cell activation markers. Leukocyte numbers and T cell proportions remained stable, which suggested activation of a resident population. Among cytotoxic granules, *GNLY* was the only one upregulated in immune horses. While its role in bacterial infection is established, fewer studies have explored its function during viral infection [57–59]. Notably, rodents lack a homolog for granulysin [60], and horses may serve as a useful model for studying its activity during viral disease. By the end of the first week post-infection, immune horses had returned to baseline gene expression. Overall, the magnitude of the immune response was lower in immune horses than in non-immune horses, with pre-existing adaptive immunity contributing to rapid viral clearance and restoration of homeostasis at the URT.

This article focuses on nasal mucosal T cells at the URT and their contributions to host immunity of immune and non-immune horses after EHV-1 infection. However, it is important to emphasize that many other cell types, both immune and structural cells, exist at the URT and/or are called to the infection site after EHV-1 enters the equine host. The local viral defense against EHV-1 is complex and requires well-orchestrated interactions of multiple cell types at each infection stage. Upon EHV-1 infection at the URT, a delicate balance of innate immune recognition, activation, control of inflammation, and induction of adaptive immune mechanisms is required for effective host immunity. The initial mucosal immune response is crucial for minimizing viral replication at the URT, primes the adaptive immune system for controlling the systemic spread of the virus, and likely majorly influences overall infection outcomes and severity of disease.

## Supplementary Information

**Additional file 1. Clinical and virological outcomes of EHV-1 infection. **Horses were intranasally challenged with 1 x 10^7^ PFU/mL of Ab4 EHV-1. Immune status was retrospectively determined based on the presence or absence of (A) fever and virus detection in (B) nasal secretions and (C) peripheral blood of the horses. Horses that were non-immune (*n *= 4, black) or immune (*n *= 4, green) were selected for RNAseq, and the results of infection for these horses is displayed. (A) Temperature was measured by rectal thermometer, and considered a fever when >38.6 ºC, indicated by the horizontal dotted line. (B) Nasal shedding was detected in nasal swab samples by plaque assay and reported as PFU/mL. (C) Viremia was detected in PBMC by an EHV-1 gB qPCR assay and reported as Ct value, with a detection cut-off of 37.38 indicated by the horizontal dotted line. Points represent mean and error bars represent SEM.

## Data Availability

The dataset supporting the conclusions of this article is available in the NCBI’s Gene Expression Omnibus (GEO) repository, https://www.ncbi.nlm.nih.gov/geo/query/acc.cgi?acc=GSE232941.
