# Supplementary material for: Mucosal T cell activation pathways are upregulated by equine herpesvirus type 1 infection
Source: Vet Res. 2026 Apr 6;57:67. doi: 10.1186/s13567-026-01741-x (PMC13154595; doi:10.1186/s13567-026-01741-x)
Supplement: Supplementary file 1 — Additional file 1. Clinical and virological outcomes of EHV-1 infection. Horses were intranasally challenged with 1 x 10^7^ PFU/mL of Ab4 EHV-1. Immune status was retrospectively determined based on the presence or absence of (A) fever and virus detection in (B) nasal secretions and (C) peripheral blood of the horses. Horses that were non-immune (n = 4, black) or immune (n = 4, green) were selected for RNAseq, and the results of infection for these horses is displayed. (A) Temperature was measured by rectal thermometer, and considered a fever when >38.6 ºC, indicated by the horizontal dotted line. (B) Nasal shedding was detected in nasal swab samples by plaque assay and reported as PFU/mL. (C) Viremia was detected in PBMC by an EHV-1 gB qPCR assay and reported as Ct value, with a detection cut-off of 37.38 indicated by the horizontal dotted line. Points represent mean and error bars represent SEM. [file 13567_2026_1741_MOESM1_ESM.docx]

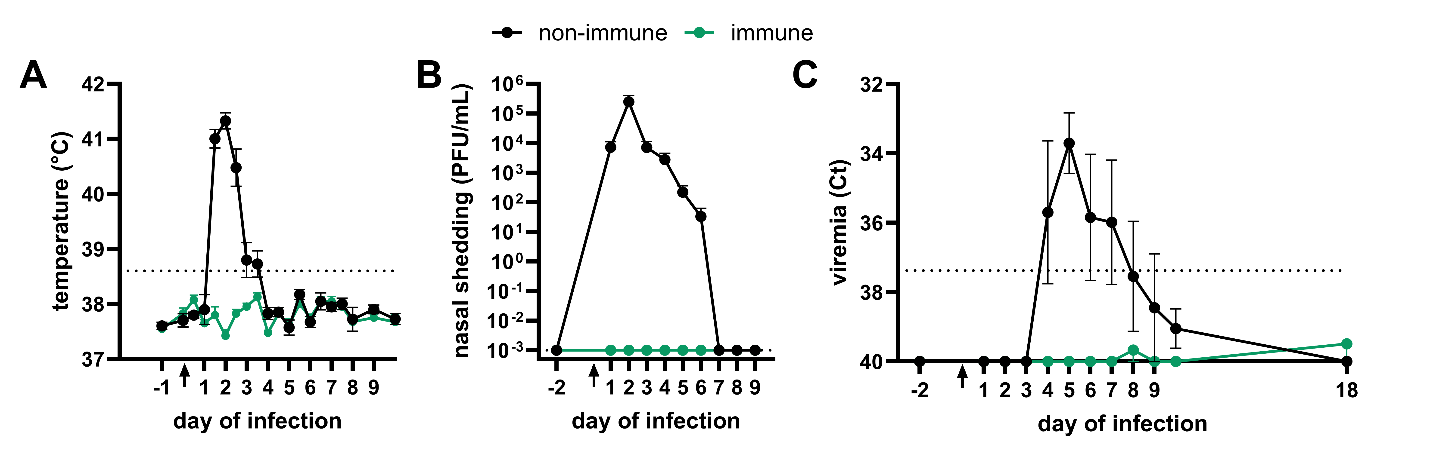


**Additional file 1: Clinical and virological outcomes of EHV-1 infection.** Horses were intranasally challenged with 1x10^7^ PFU/mL of Ab4 EHV-1. Immune status was retrospectively determined based on the presence or absence of (A) fever and virus detection in (B) nasal secretions and (C) peripheral blood of the horses. Horses that were non-immune (n=4, black) or immune (n=4, green) were selected for RNAseq, and the results of infection for these horses is displayed. (A) Temperature was measured by rectal thermometer, and considered a fever when >38.6 ºC, indicated by the horizontal dotted line. (B) Nasal shedding was detected in nasal swab samples by plaque assay and reported as PFU/mL. (C) Viremia was detected in PBMC by an EHV-1 gB qPCR assay and reported as Ct value, with a detection cut-off of 37.38 indicated by the horizontal dotted line. Points represent mean and error bars represent SEM.
